# Tyree's Antiseptic Powder

**Published:** 1904-11

**Authors:** C. F. Taylor

**Affiliations:** the Medical World.


### TYREE'S ANTISEPTIC POWDER.

Dr. C. F. Taylor, in the *Medical World.*

After an inspection of the process originated and employed by the manufacturer of this preparation, we do not wonder at its phenomenal success, and as an item of interest to the medical profession, we copy it in detail.

One hundred pounds of borax and one hundred pounds of alum are placed in a crucible of proper size and fused together with heat. The water of crystallization will be driven off, and the borax and alum will fuse together in a mass. When taken from the crucible it occurs in large lumps, which must be reduced to a powder by means of appropriate machinery. Then the powder is passed through sieves of graduated degrees of fineness, in order to get the desired fineness and a uniform degree of fineness. Then antiseptics and aromatics are added to the powder, the manipulations being done by appropriate machinery. When this is completed, we have "Tyree's Pulv. Antiseptic Comp.," or, for short, Tyree's Antiseptic Powder.

You see the impossibility of making this up as an extemporaneous prescription. You see, also, that to make it properly requires special apparatus, facilities, etc. Besides, it requires about five days to complete a "batch" of it. Even if your corner druggist was prepared to make it properly your patient could not wait five days to have the prescription filled. So the proper thing to do is to get it from headquarters, or have your druggist to carry it in stock, in order to fill your prescriptions.

---

Aluminum Lining For Rubber Plates.—A simple method is as follows: Take chloroform, three ounces; carbon disulphid, one ounce; powdered aluminum, one ounce. Before mixing the ingredients make a saturated solution of the chloroform and white vulcanite rubber. When the case is ready to pack, give the model a good coat of collodion, and follow this with about three or four coats of the aluminum preparation. I have used this for about six years, and have one plate under observation made in this way five years ago, and can see no change in it.—Dr. Burdette L. Conway, Dental Brief.

Chloropercha.—In making the solution, after you have applied the chloroform, stir up and add eucalyptol until you have it in a creamy state. Then leave the cork out of the bottle to allow the chloroform to evaporate. You then have a solution which, if forced through the apical foramen, will cause less irritation than would the chloroform.—Dr. C. S. Caesar, Review.

Treatment of Dry-Socket.—A patient for whom a third molar had been extracted by a specialist returned two days afterwards for consultation,. complaining of extreme pain in the face, and of the teeth in both maxillae simulating irritation of a pulp. Careful examination excluded the teeth. When the alvelus from which the tooth had been removed was examined, it was found devoid of the normal coagulum, with the bone bare. A stiff dough-like paste, similar to a suppository, was made of orthoform, combined with oil of sesame and glycerin, with which the socket was filled. This means gave protection to the exposed tissue, as well as nearly immediate relief by the analgesic properties of the orthoform. —International.

